# Incidence, risk factors, and outcomes in electroencephalographic seizures after mechanical circulatory support: A systematic review and meta-analysis

**DOI:** 10.3389/fcvm.2022.872005

**Published:** 2022-08-03

**Authors:** Qian Li, Jingjia Shen, Hong Lv, Yue Liu, Yuye Chen, Chenghui Zhou, Jia Shi

**Affiliations:** State Key Laboratory of Cardiovascular Disease, Department of Anesthesiology, Fuwai Hospital, National Center for Cardiovascular Diseases, Chinese Academy of Medical Sciences and Peking Union Medical College, Beijing, China

**Keywords:** mechanical circulatory support, electroencephalographic seizures, cardiopulmonary bypass, extracorporeal membrane oxygenation, cardiac surgical procedures (MeSH)

## Abstract

**Purpose:**

To estimate the overall incidence, risk factors, and clinical outcomes of electroencephalographic (EEG) seizures for adults and children after mechanical circulatory support (MCS).

**Method and measurements:**

This systematic review and meta-analysis were carried out in accordance with the PRISMA (Preferred Reporting Items for Systematic Review and Meta-Analysis) guidance document. MEDLINE EMBASE and CENTRAL were investigated for relevant studies. The related information was retrieved by two independent reviewers and all analyses were conducted by STATA (version 16.0; Stata Corporation, College Station, TX, United States).

**Result:**

Sixty studies including 36,191 adult and 55,475 pediatric patients with MCS were enrolled for evaluation. The study showed that the overall incidence of EEG seizures in adults was 2% (95%CI: 1–3%), in which 1% (95%CI: 1–2%) after cardiopulmonary bypass (CPB), and 3% (95%CI: 1–6%) after extracorporeal membrane oxygenation (ECMO). For pediatrics patients, the incidence of EEG seizures was 12% (95%CI: 11–14%), among which 12% (9–15%) after CPB and 13% (11–15%) after ECMO. The major risk factors of EEG seizures after MCS in adults were redo surgery (coefficient = 0.0436, *p* = 0.044), and COPD (coefficient = 0.0749, *p* = 0.069). In addition, the gestational week of CPB (coefficient = 0.0544, *p* = 0.080) and respiratory failure of ECMO (coefficient = –0.262, *p* = 0.019) were also indicated to be associated with EEG seizures in pediatrics.

**Conclusion:**

EEG seizures after MCS were more common in pediatrics than in adults. In addition, the incidence of EEG seizure after ECMO was higher than CPB both in adults and children. It is expected that appropriate measures should be taken to control modifiable risk factors, thus improving the prognosis and increasing the long-term survival rate of MCS patients.

**Systematic Review Registration:**

[https://www.crd.york.ac.uk/prospero], identifier [CRD42021287288].

## Introduction

With an increasing number of cardiac surgeries globally, a large number of elderly patients and children require mechanical circulatory support (MCS) during cardiac surgery. Epidemiologic studies predicted that these procedures will further pose a unique threat in the near future (1). However, accumulating evidence has shown that the use of continuous cardiopulmonary bypass (CPB) is not entirely safe, and may be associated with central nervous system injury and adverse developmental sequelae, studies reported that adult neurocognitive declined dramatically after CPB (2). Furthermore, the use of CPB or extracorporeal membrane oxygenation (ECMO) carries an inherent risk of complications and may lead to neurological decline (3–6), such as ischemic and hemorrhagic stroke and seizures, which are associated with prolonged hospital stays and increased risk of poor outcome (5).

Seizures are defined as the transient occurrence of signs or symptoms due to abnormal excessive or synchronous neuronal activity in the brain (7). Clinical diagnostics require experience and professional personnel. As patients with MCS typically require sedation, the neurological examination is not always reliable, but neuro-monitoring is essential to the success of cardiovascular surgery. Apart from clinical signs, the only confirmatory tool for diagnosis is electroencephalography (EEG) (8), which can be performed intermittently or continuously (9). Early identification of seizures could mitigate brain injury and reduce mechanical ventilation duration, intensive care unit, and hospital stay (10, 11). EEG is a non-invasive tool that measures cortical electrical activity, with an excellent spatial and temporal resolution and sensitivity to changes in both brain structure and function, which plays an indispensable role in diagnosing seizures (12). The EEG can not only provide valuable insights into seizure monitoring but also evaluate adverse neurological outcomes. Furthermore, most subclinical seizures have no discernible clinical correlation and could result in underestimation without continuous EEG monitoring (13). Accumulating evidence has revealed that the identification of subclinical seizures is essential for the prevention of MCS morbidity following cardiac surgery. Concern was expressed about the incidence of EEG seizures following MCS. However, there is no systematic review regarding the incidence of EEG seizures in adult and pediatric patients following MCS.

Therefore, the primary purpose of this systematic review and meta-analysis was to explore the incidence and prognostic effect of EEG seizures after MCS in adult and pediatric patients. Besides, the potential risk factors of EEG seizures in adult and pediatric patients after MCS were also investigated.

## Methods

### Search strategy and selection criteria

A systematic review and quantitative meta-analysis of published studies examining the incidence of EEG seizures following MCS was conducted. This systematic review was conducted following the Preferred Reporting Items for Systematic Reviews and Meta-analysis (PRISMA) and registered in the prospective international register of systematic reviews (PROSPERO) (registration number is CRD42021287288). MEDLINE, EMBASE, and CENTRAL databases were comprehensively searched for relevant studies from inception to 25 November 2021. Electroencephalography, seizures, CPB, ECMO, and all synonyms related to the terms were included. Furthermore, references from related reviews and studies were manually searched. Details of research strategies are provided in Supplementary File 1.

All retrieved studies were independently identified by two investigators (QL and JJ S) for potential eligibility. The titles, abstracts, and full texts of these articles were analyzed to select studies for inclusion in this systematic review. The inclusion criteria were: (1) adults (≥18 year of age) or pediatric (≤18 year of age) who underwent mechanical circulatory (CPB OR ECMO) during cardiac surgery; (2) the seizures were diagnosed by EEG; (3) the incidence of seizures was recorded; (4) cohort studies or randomized control trials (RCTs); (5) language was restricted in English. On the other hand, studies that were case reports, animal design, conference abstracts, previous reviews, books, theses, or editorial letters were excluded. We also excluded articles that do not clearly illustrate the incidence of EEG seizures or the MCS records.

### Data extraction and quality assessment

The following information from eligible studies was extracted by two reviewers independently: study information, including the authors’ names, study design, year, country, type of mechanical support; preoperative population demographics (gender, age, and complications), perioperative information (duration of CPB, duration of ECMO, duration of DHCA, duration of aortic clamp time, surgery type), postoperative data (length of hospital stay and duration of mechanical ventilation); sample size; the incidence of EEG seizure.

The Newcastle-Ottawa Quality Assessment Scale (NOS) was used for quality assessment. Since our goal is to explore the incidence of EEG seizures following MCS, minor modifications, the removal of two elements from the control group, have been made to the NOS scale. A total of six items were included in the amended quality evaluation. Details are available in Supplementary File 2.

Any disagreement was resolved by discussion between the two evaluators.

### Endpoints

The primary endpoint was the incidence of EEG seizures following MCS. Secondary endpoints were outcomes and risk factors of EEG seizures during the surgical period for follow-up assessment and the mortality rate during this period.

### Statistical analysis

The incidence of EEG seizures after MCS in adults and pediatrics was calculated by the Stata (Version 16.0; Stata Corporation, College Station, TX, United States). Heterogeneity between studies was evaluated by Cochran Q and I2 statistics with a threshold of *p* < 0.1 to determine heterogeneity, and *I*^2^ values >50% indicated heterogeneity. Taking into account the heterogeneity between studies, the random-effect model was used to estimate the mean effect and accuracy, and to provide a more conservative estimate of the 95% CI.

The potential publication bias was evaluated by Begg and Egger test. The trim-and-filled method was used in case of significant publication bias. Sensitivity analyses were conducted by removing each included study at one time to obtain the remaining overall estimate of EEG seizure incidence. In addition, meta-regression (*p* < 0.1) and subgroup analysis were conducted to detect potential sources of significant heterogeneity. The identification of each factor in the meta-regression analyses was considered in at least four studies.

## Result

### Study characteristic

**FIGURE 1:**
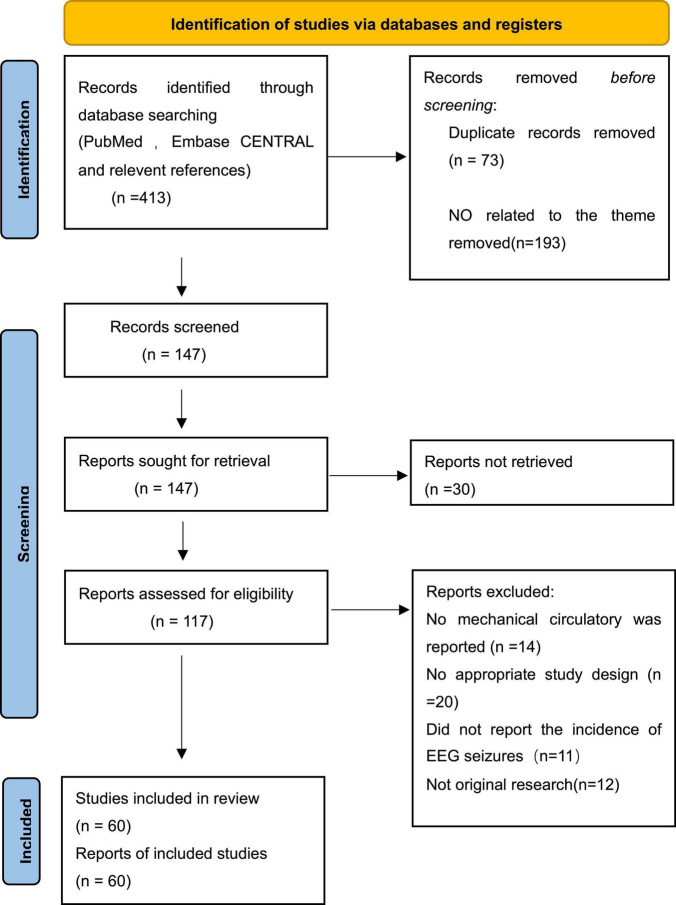
Flowchart for complete screening, including process.

A total of 413 potentially pertinent studies were identified by a systematic search of related databases. After 296 abstracts were excluded from the restricted search due to duplicated publication (*n* = 73), irrelevant (*n* = 193), and not retrieved the reports (*n* = 30), then 117 published papers met the inclusion criteria for the full-text review. In addition, 57 published papers were excluded for the following reasons: mechanical circulatory support was not reported (*n* = 14); the incidence of EEG seizure was not reported (*n* = 11); inappropriate study design (*n* = 20); no original research (*n* = 12). Finally, sixty studies were retained for final evaluation and quantitative analysis. Supplementary File 3 shows the characteristics of the included studies, and Figure 1 shows the diagram of the study selection.

### Quality assessment

Based on the modified NOS scale, 9 in 13 studies of the adult population and 31 in 47 studies of the pediatric population scored 4 points or more, demonstrating low or moderate risks of bias. Two studies reported both the adult and pediatric population (14, 15), and the sample size of some studies is relatively small and the reporting year is from 1974 to 2021, which may be attributed to the selection bias. The second bias derives primarily from the evaluation of outcomes. EEG monitoring should not be used in all patients, and the incidence of EEG seizures may be underestimated. Besides, not all the periods and the adequacy of follow-up were demonstrated in enrolled studies (Supplementary File 2).

### Effect of mechanical circulatory support on the incidence of electroencephalographic seizures in the adult and pediatric population

**FIGURE 2:**
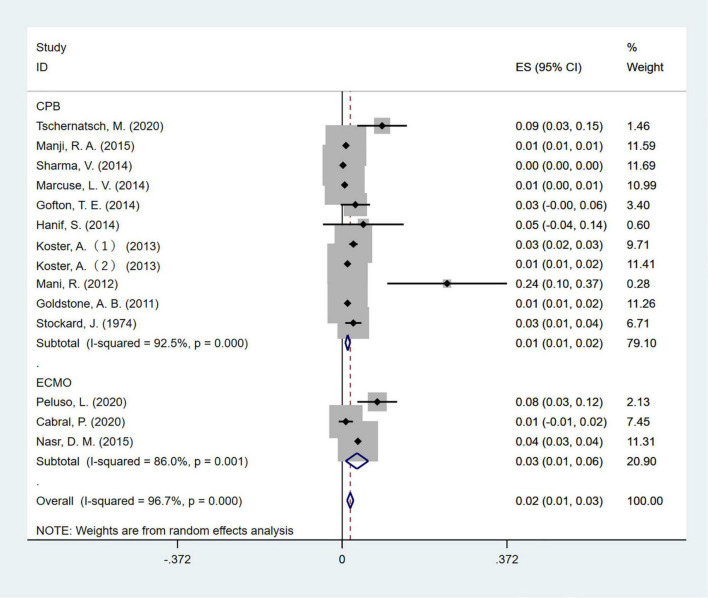
The forest plot shows the pooled event rate with 95% CIs of all electroencephalography seizures after mechanical support in adults, the random-effects model was used.

**FIGURE 3:**
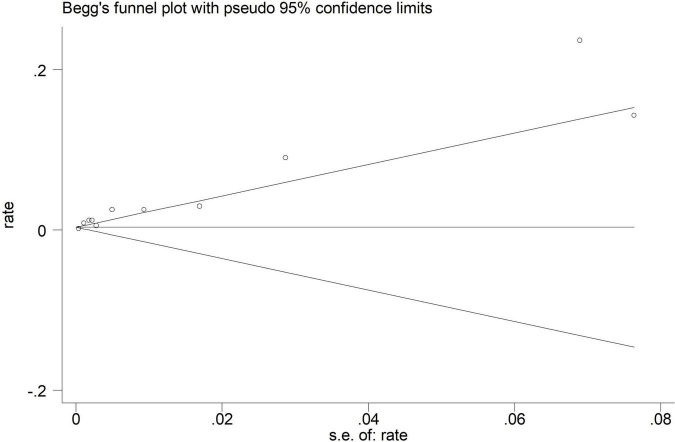
Begg’s funnel plot analysis of cardiopulmonary bypass (CPB) in adults.

**FIGURE 4:**
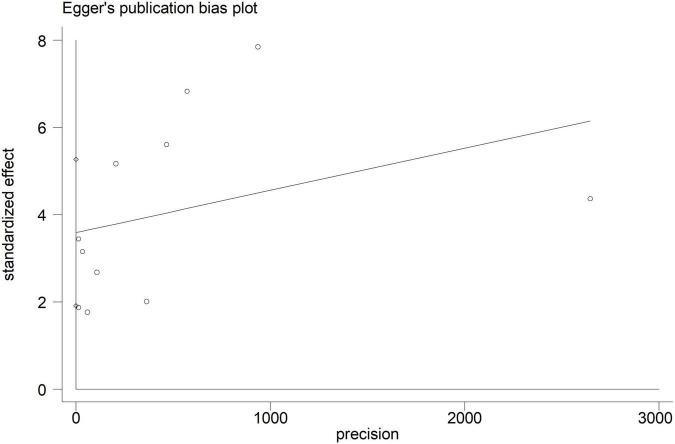
Egger’s funnel plot analysis of cardiopulmonary bypass (CPB) in adults.

Electroencephalographic (EEG) seizure after MCS was reported in 36,191 study adult subjects, and the overall incidence was 2% (95%CI: 1–3%), in which the incidence of EEG seizures was 1% (95%CI: 1–2%) with CPB and 3% (95%CI: 1–6%) with ECMO, separately (Figure 2). Study heterogeneity was significant, with an overall *I*^2^ of 96.7%. In addition, a potential evident publication bias was observed in adults with CPB (Egger test: *p* = 0.001; Begg test: *p* = 0.533; Figures 3, 4). The trim-and-filled method further confirmed the robustness of the EEG seizure in adults with CPB (*P* < 0.001).

**FIGURE 5:**
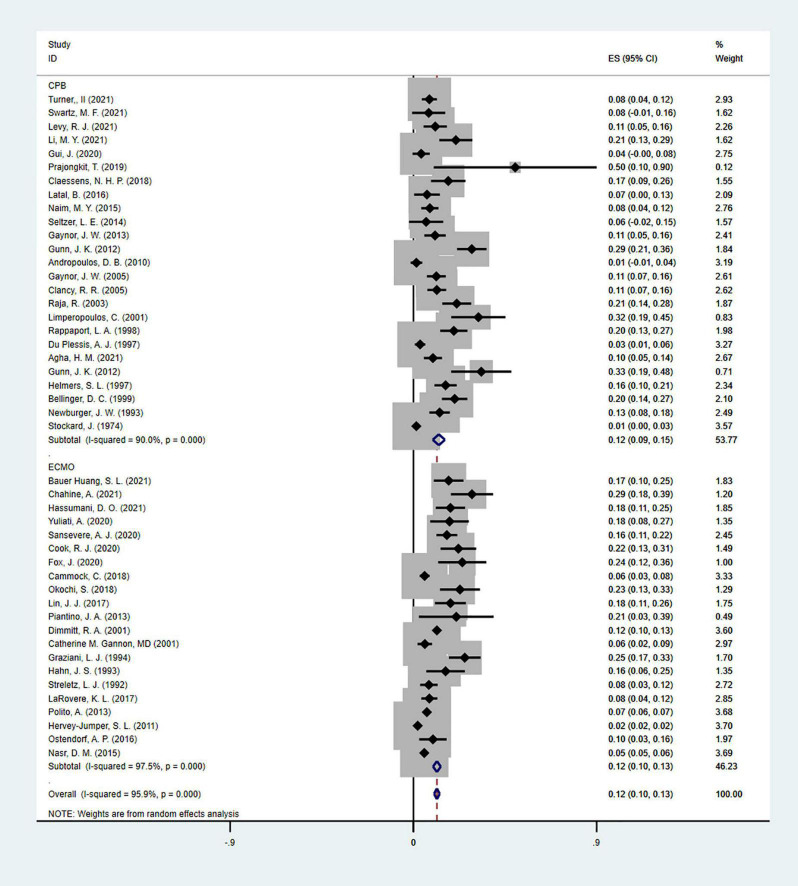
The forest plot of the incidence of electroencephalographic (EEG) seizures after mechanical support in pediatrics.

**FIGURE 6:**
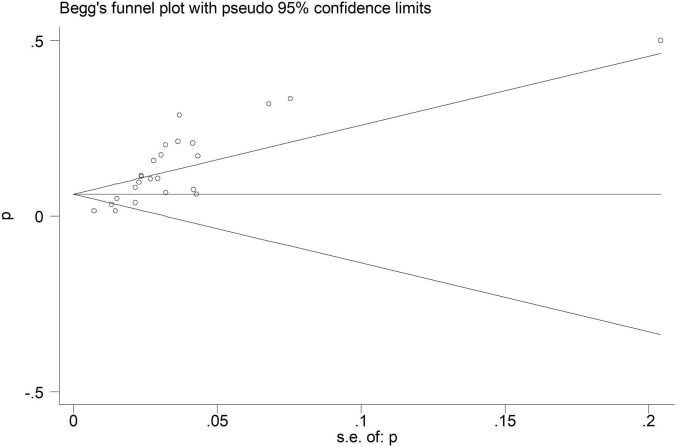
Begg’s funnel plot analysis of cardiopulmonary bypass (CPB) in pediatrics.

**FIGURE 7:**
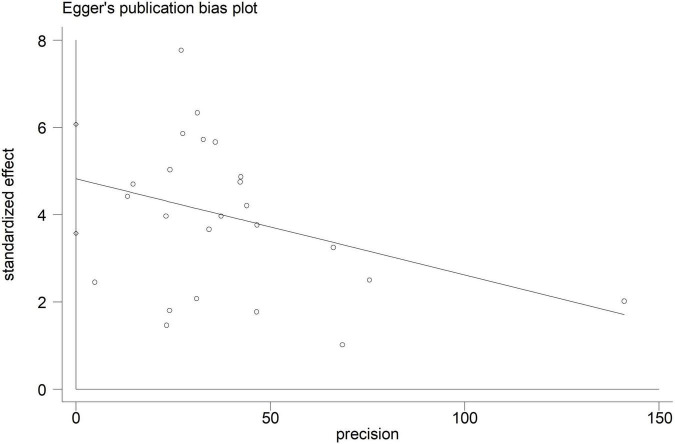
Egger’s funnel plot analysis of cardiopulmonary bypass (CPB) in pediatrics.

**FIGURE 8:**
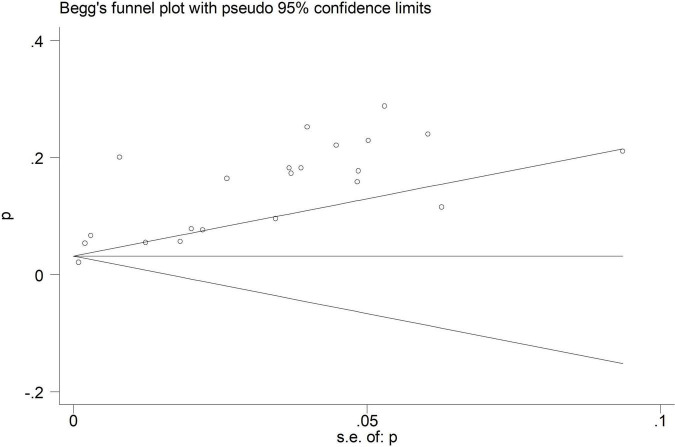
Begg’s funnel plot analysis of extracorporeal membrane oxygenation (ECMO) in pediatrics.

**FIGURE 9:**
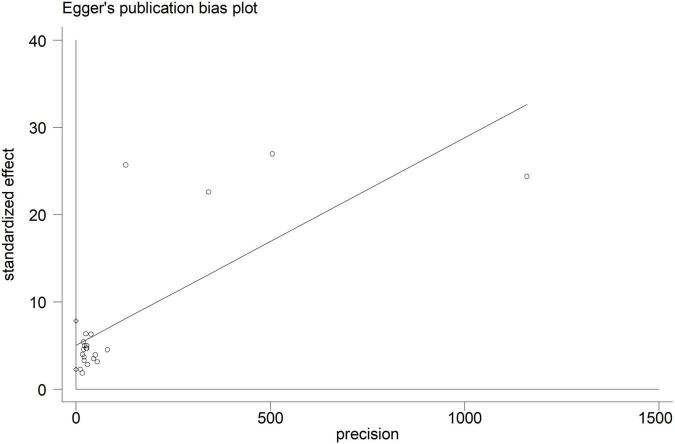
Egger’s funnel plot analysis of extracorporeal membrane oxygenation (ECMO) in pediatrics.

Moreover, EEG seizures after MCS were reported in 55,475 pediatric subjects, and the overall incidence was 12% (11–14%), in which the incidence of EEG seizures was 12% (9–15%) with CPB, and 13% (11–15%) with ECMO, respectively (Figure 5). Study heterogeneity was significant, with an overall *I*^2^ of 95.8% for pediatrics. In addition, a potential evident publication bias was observed in pediatrics with CPB (Egger test: *p* < 0.001; Begg test: *p* = 0.001; Figures 6, 7) and ECMO (Egger test: *p* = 0.001; Begg test: *p* = 0.693; Figures 8, 9). The trim-and-filled method further confirmed the robustness of the EEG seizure in pediatrics with MCS (*P* < 0.001).

### Potential sources of significant heterogeneity: sensitivity analysis, meta-regression, and subgroup analysis

**FIGURE 10:**
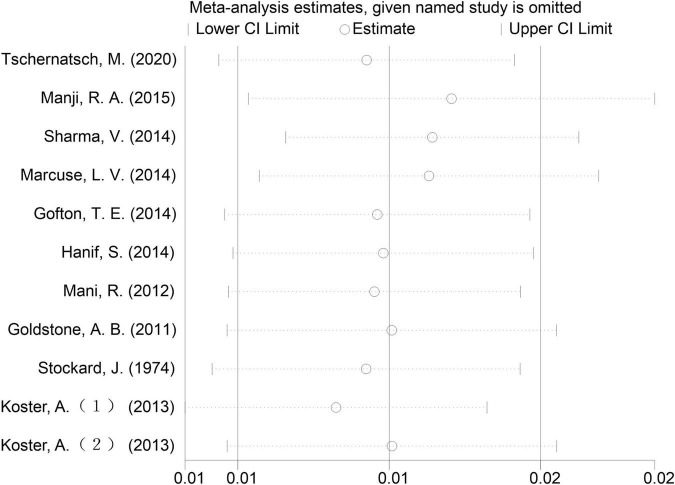
Influence analysis of cardiopulmonary bypass (CPB) in adults.

**FIGURE 11:**
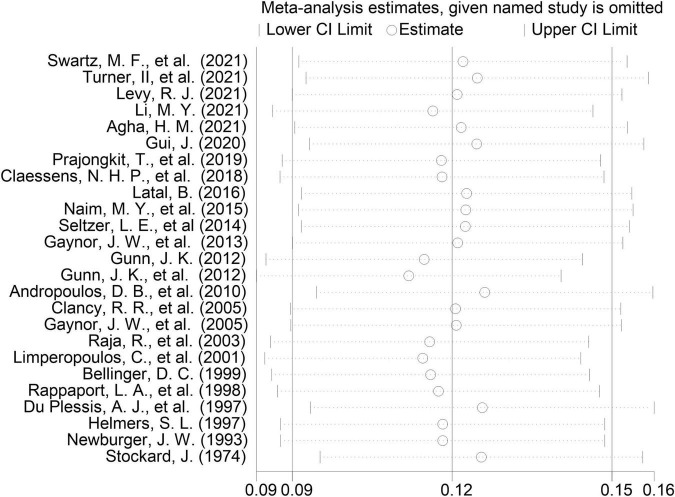
Influence analysis of cardiopulmonary bypass (CPB) in pediatrics.

**FIGURE 12:**
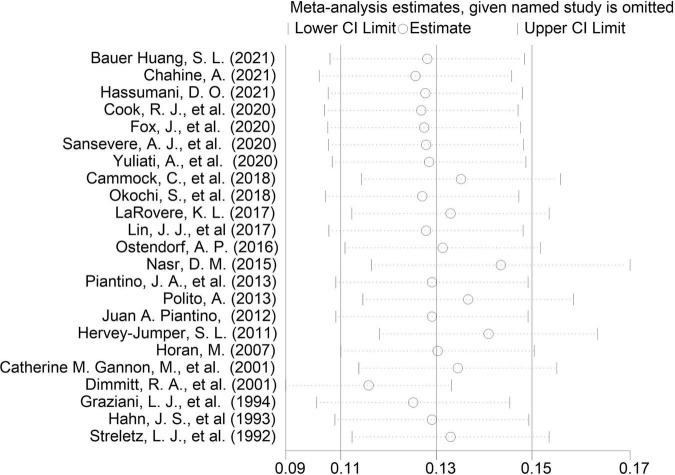
Influence analysis of extracorporeal membrane oxygenation (ECMO) in pediatrics.

A sensitivity analysis that ruled out each study at one time revealed that no single study affected the overall effect size of EEG seizures after MCS dramatically in adults and pediatrics (Figures 10–12).

### Adults

Age, female, diabetes, hypertension, myocardial infarction, chronic obstructive pulmonary disease (COPD), peripheral vascular disease, stroke, redo surgery, extracorporeal circulation time, aorta clamp time, coronary artery bypass graft (CABG), valvular surgery, combined CABG, and valvular surgery, emergency or urgent surgery were included in the random-effect univariate meta-regression analysis for EEG seizure after CPB in adults. As a result, the identified major sources of significant heterogeneity were redo surgery (coefficient = 0.0436, *p* = 0.044, adjusted *R*^2^ = 78.69%), and COPD (coefficient = 0.0749, *p* = 0.069, adjusted *R*^2^ = 58.07%) as shown in Table 1. Furthermore, subgroup analysis showed that adult subjects with a mean age of <65 years old had a lower risk of EEG seizures after CPB than those with a mean age of >65 years old (*p* < 0.0001 for subgroup difference; Table 2). In addition, studies without redo surgery and COPD had significantly lower EEG seizures after CPB than those with redo surgery or COPD (*p* < 0.0001 for subgroup difference; Table 2).

**TABLE 1:** Meta-regression analysis in adults and pediatrics.

Factors	No. of studies	Coefficient	*P*-value	95% CI	Adjusted R^2^(%)
**Adult**					
Redo surgery	6	0.0436094	0.044	(0.0018729, 0.085346)	78.69
COPD	6	0.0749121	0.069	(–0.0094428, 0.159257)	58.07
**Pediatric**					
CPB gestational age week	16	0.0543747	0.080	(–0.007398, 0.1161473)	29.02
ECMO criteria-respiratory	8	–0.2615983	0.019	(–0.4619913, –0.0612051)	72.91

**TABLE 2:** Subgroup analysis for adult and pediatric.

Factors	Q0	Q1	Q2	Qall	p
**Adult**					
Age	4.21	37.67	14.6	135.22	<0.001
COPD		25.24	79.28	135.22	<0.001
Redo surgery		25.24	79.28	135.22	<0.001
**Pediatric**					
Gestation week (CPB)	82.52	7.12	44.17	241.71	<0.001
Respiratory (ECMO)		825.80	68.37	1090.19	<0.001

### Pediatrics

Female, gestational age (week), birth weight, birth head circumference (cm), Apgar score, weight at operation, age at operation, total CPB time (min), total support time (min), duration of cumulative DHCA time (min), DHCA (%), aortic cross clamp time (min), delayed sternal closure (%) were included in the random-effect univariate meta-regression analysis for EEG seizure after CPB in pediatrics. As a result, the identified major sources of significant heterogeneity were gestational age (week) (coefficient = 0.0544, *p* = 0.080, adjusted *R*^2^ = 29.02%) as shown in Table 1. Subgroup analysis indicated that pediatric subjects with a median gestational age (week) < 38.5 had a lower risk of EEG seizures after CPB than those with a median gestational age (week) > 38.5 (*p* < 0.0001 for subgroup difference; Table 2).

Female, gestational age (week), weight at operation, ECMO criteria: extra-cardiopulmonary resuscitation (E-CPR), cardiac, respiratory, ECMO type [venous-venous (VV) or venous-artery (VA)], ECMO duration, and the cannulation site (neck, central, and femoral) were included in the random-effect univariate meta-regression analysis for EEG seizure after ECMO in pediatrics. As a result, the identified major source of significant heterogeneity was ECMO criteria-Respiratory (coefficient = –0.2616, *p* = 0.019, adjusted *R*^2^ = 72.911%) as shown in Table 1. Subgroup analysis indicated that pediatric subjects without respiratory failure had a lower risk of EEG seizures after ECMO than those with respiratory failure (*p* < 0.0001 for subgroup difference; Table 2).

### Prognostic effect of electroencephalographic seizure on outcomes

#### Length of hospital stay

There was no significant increase in hospital stay in the EEG seizures group compared to the no EEG seizures group [weighted mean difference (WMD) = 4.2 days; 95%CI: –2.499 to 10.931; *P* = 0.218] (16, 17). Meanwhile, no statistical significance was detected in the EEG seizures group after ECMO (WMD: 3.301 days; 95%CI: –20.072 to 26.673; *p* = 0.782) (6, 18) in pediatric. However, a dramatic increase was observed in the group of EEG seizures after CPB as compared with the no EEG seizures group (WMD = 8.8 days; 95%CI: 1.3–16.385; *P* = 0.022) in pediatric (19–22).

#### Length of intensive care unit stay

A significant increase in length of intensive care unit (ICU) stay was observed in the group of EEG seizures after CPB as compared with the no EEG seizures group both in adults (WMD = 5.2 days; 95% CI: 1.908–8.475; *P* = 0.002) (17, 23, 24) and pediatrics (WMD = 7.1 days; 95% CI: 3.239–10.965; *p* < 0.001) (20, 21).

#### Hospital mortality

The analysis we performed suggested that the incidence of hospital mortality of EEG seizures in adults after CPB support was 4.5 times that of no EEG group (OR: 4.51 [2.91–6.98], *p* < 0.001) (16, 17, 24, 25).

#### Ventilation support

As for ventilator support, a trend of increase in the EEG seizures group in adults was observed as compared with the no EEG seizures group (WMD = 3.4 days; 95% CI: –0.305 to 7.157, *p* = 0.072) (17, 24). However, no significant increase was detected in the EEG seizures group of pediatrics after CPB (WMD = 3.2 days; 95%CI: –2.536 to 8.874; *p* = 0.276) (19–21).

#### Duration of cardiopulmonary bypass or extracorporeal membrane oxygenation

Statistically significant was observed in the duration of CPB or ECMO in the EEG seizures group of pediatrics compared with no EEG seizures group (WMD = 26.595 min; 95%CI: 11.584–41.605; *p* = 0.001) (22, 26, 27).

## Discussion

In this systematic review and meta-analysis of 60 studies involving 36,191 adults and 55,475 pediatric patients, the results showed that the incidence of EEG seizures after MCS was 2% (95% CI: 1–3%) in adults and 12% (11–14%) in pediatrics.

To our knowledge, there is no quantitative analysis of the incidence, clinical outcomes, and risk factors of EEG seizures after MCS in different populations, making the results of this analysis more accurate and more convincing than the previous qualitative studies. Regarding risk factors of EEG seizures after MCS, we summarized age, COPD, and redo-surgery were related to the EEG seizures after MCS in adults. Furthermore, gestational week and respiratory failure were found to be associated with EEG seizures after MCS in pediatric patients. In addition, it is also worth noting that the clinical outcomes of EEG seizures were worse than the group without EEG seizures both in adults and pediatrics after MCS.

With the increasing attention and applications of MCS recently, patients with MCS have improved clinical outcomes, but there are some possible neurological complications it may bring, such as seizures, ischemic strokes, and intracranial hemorrhage, which may frustrate its benefit (28). Neuromonitoring plays a crucial role in enhancing a wide range of MCS neurological outcomes, with early detection and appropriate intervention (29, 30). However, the standard monitoring for MCS patients remains sparse. There are several neurological monitoring approaches in clinical practice, such as transcranial Doppler (TCD), near-infrared spectroscopy (NIRS), neuroimaging, serum injury biomarkers, and intracranial pressure (ICP) monitoring, somatosensory-evoked potentials (SEP), serial head ultrasounds (HUS) and EEG (31, 32). However, the validity of the approaches in monitoring MCS patients was inadequate because the previous studies were pilot studies with relatively small sample sizes. Thus, more validation and generalizability of neurological monitoring in MCS patients were supposed to be conducted.

The incidence of seizures was correlated with higher mortality and unfavorable neurodevelopmental outcomes (33). Therefore, great importance should be attached to the application of non-invasive monitoring (34), EEG, featuring with prompt detection of seizures for all patients with MCS (35). As for the risk factors of EEG seizures after MCS in adults, this study confirmed that age and redo surgery were correlated with EEG seizures (16, 17, 25). Besides, COPD was detected to be the risk factor for EEG seizures after CPB. The presence of COPD was considered to be a high-risk factor for CABG surgery, with detrimental postoperative outcomes (36). What is more, a retrospective study demonstrated that COPD was a risk factor for seizures in stroke patients, which may be attributed to its recurrence of oxygen desaturation at night (37). Moreover, CPB could trigger inflammatory activity, which plays a crucial role in deteriorating pulmonary and systemic. Thus, attention should be paid to the potential risk factors of EEG seizure with MCS. Since seizures are potentially treatable with early detection and medications, therefore, appropriate treatment of early postoperative seizures may be of high clinical importance.

Concerning the risk factors of EEG seizures after MCS in pediatrics, previous studies reported that EEG seizures were more common in infants undergoing circulatory arrest (38). In this study, we confirmed that gestational age was correlated with EEG seizures after CPB. Furthermore, respiratory failure was observed to be the primary risk factor for EEG seizures after ECMO in pediatrics. ECMO can serve as an artificial lung, which can provide an extracorporeal gas exchange to maintain adequate oxygenation and carbon dioxide removal in the acute phase of respiratory (39, 40). Furthermore, the V-V ECMO was commonly utilized in respiratory pediatric (41–43). However, the initiation of ECMO can activate the release of cytokines and lead to vasoplegia, which may facilitate coagulation abnormalities and precipitate ischemic and hemorrhagic injuries (44, 45). Therefore, the respiratory condition before and during surgery should be of great importance, and measures should be taken to protect the respiratory system of pediatrics, whose lungs are immature and more susceptible to the influence of ECMO. Conspicuously, the identification of risk factors can help to stratify patients and improve the management and prognosis of pediatrics.

In terms of clinical outcomes, this study suggested that the hospital mortality and ventilation support time of EEG seizures after CPB were worse than the group without EEG seizures in adults. Furthermore, in adults, EEGseizures following CPB were significantly associated with mortality (14, 46) In addition, the length of hospital stays and the duration of CPB or ECMO in the EEG seizures group after CPB were worse than in the group without EEG seizures in the pediatric patients. Moreover, the length of ICU stays was longer in the EEG seizures group in adults as well as in pediatrics.

In this study, we found obvious differences in a diverse population (adult vs. pediatric patients) and types (CPB vs. ECMO) of MCS. Although the related precise underlying mechanisms remain unclear, the following reasons could not be ruled out: first, the pediatric population is more susceptible to the impact of MCS. In that, the organ structure and the immune system of the child are more premature. Furthermore, the neonates were non-verbal and they could not express the sensory phenomena linked with seizures, thus the majority of neonatal seizures were ascertained by EEG (47). Moreover, the congenital heart disease is better to be repaired in the neonatal and infant period, which may be more accessible to the hemodynamic instability resulting from the systemic inflammatory response of MCS, leading to an increased vulnerability to brain injury (48). Second, CPB is conducted at a low temperature while ECMO is mostly at a normal temperature. It is reported that low temperature has a certain brain protection ability (49). What is more, there are some differences between CPB and ECMO. CPB provides complete respiratory support and hemodynamic stability along with the ability to manipulate flow and blood pressure according to the requirements of certain stages of the operation, but it may activate systematic inflammation. Venous-venous (VV) ECMO offers breathing support without hemodynamic support. And venous-arterial (VA) ECMO provides respiratory and hemodynamic support (50), whereas intraoperative ECMO can result in hemodynamic instability, catastrophic air embolism, and other complications (50). Seizures of EEG in adults following MCS, however, should be given more prominence, which is more likely to be overlooked in clinical practice. Although the literature is sparse on the use of EEG monitoring in adult patients on ECMO (46), Interestingly, the incidence of EEG seizures in adult ECMO patients was quite limited, as antiepileptic drugs were rapidly initiated to limit these events, which should be paid more attention to.

Previous studies showed that EEG seizures occur more frequently than clinical seizures, highlighting the importance of clinicians using EEG to detect subclinical seizures. The actual incidence of postoperative seizures is probably underestimated, especially since detection is difficult and diagnosis is often based solely on clinically apparent signs and symptoms. As a result, specially trained clinicians will report seizure activity more accurately than a person who does not have this type of clinical routine or a researcher who consults a hospital database. Because the number of patients with multiple comorbidities undergoing cardiac surgical procedures will likely increase in the future, it could be assumed that the incidence of neurologic complications, including seizures, will rise concomitantly. All mechanisms leading to seizure activity in adults following cardiac surgery have not been determined. As well, the prevalence of EEG seizures associated with MCS appears to be multifactorial. Risk factors associated with seizures, most of which cannot or may only be partially altered. In other words, early diagnosis of seizures and identification of underlying causes is essential to reduce EEG seizure-associated long-term morbidity and mortality of patients (51).

There are several limitations to this review we need to illustrate. First, this meta-analysis is quite heterogeneous after a comprehensive analysis, but we have elaborated on the possible reasons for the heterogeneity, sensitivity analysis, meta-regression, and subgroup analysis were used to explore the potential sources of heterogeneity. Second, it was not possible to distinguish between patients receiving venous-arterial and venous ECMO, except for certain studies that demonstrated practical methods. Third, we could not rule out the potentially harmful effect of publication bias on the results. Fourth, EEG provides data that helps clinicians in decision-making in the setting of seizures-related situations (52). However, not all patients have access to EEG follow-up, which may lead to a potential underestimation of the real incidence of EEG seizures.

## Conclusion

EEG seizures after MCS are more common in pediatrics than in adults. In addition, the incidence of EEG seizures after ECMO was higher than CPB, both in adults and children. It is expected that appropriate measures should be taken to control modifiable risk factors, thus improving the prognosis and increasing the long-term survival rate of MCS patients.

## Data availability statement

The original contributions presented in this study are included in the article/Supplementary material, further inquiries can be directed to the corresponding authors.

## Author contributions

QL contributed to the whole process of the manuscript, including the search strategy conceptualization and writing—original draft preparation. JJS and YL contributed to the software and helped in the investigation. HL and YC coordinated the systematic review. CZ and JS contributed to reviewing and editing. CZ contributed to the visualization. JS contributed to the supervision and administration of the project. All authors contributed to the article and approved the submitted version.

## Supplementary material

Supplementary File 1 Search strategy.

Supplementary File 2 Risk of bias in enrolled studies.

Supplementary File 3 Characteristic of enrolled studies.

## References

[1] Alkhatip A Kamel MG Farag EM Elayashy M Farag A Yassin HM Deep hypothermic circulatory arrest in the pediatric population undergoing cardiac surgery with electroencephalography monitoring: A systematic review and meta-analysis. *J Cardiothorac Vasc Anesth.* (2021) 35:2875–88. 10.1053/j.jvca.2021.01.039 33637420

[2] Newman MF Kirchner JL Phillips-Bute B Gaver V Grocott H Jones RH Longitudinal assessment of neurocognitive function after coronary-artery bypass surgery. *N Engl J Med.* (2001) 344:395–402. 10.1056/NEJM200102083440601 11172175

[3] Zangrillo A Landoni G Biondi-Zoccai G Greco M Greco T Frati G A meta-analysis of complications and mortality of extracorporeal membrane oxygenation. *Crit Care Resusc.* (2013) 15:172–8. 23944202

[4] Barrett CS Bratton SL Salvin JW Laussen PC Rycus PT Thiagarajan RR. Neurological injury after extracorporeal membrane oxygenation use to aid pediatric cardiopulmonary resuscitation. *Pediatr Crit Care Med.* (2009) 10:445–51. 10.1097/PCC.0b013e318198bd85 19451851

[5] Mehta A Ibsen LM. Neurologic complications and neurodevelopmental outcome with extracorporeal life support. *World J Crit Care Med.* (2013) 2:40–7. 10.5492/wjccm.v2.i4.40 24701415 PMC3953870

[6] Hassumani DO Shan M Mastropietro CW Wing SE Friedman ML. Seizures in children with cardiac disease on extracorporeal membrane oxygenation. *Neurocritical Care.* (2021) 36:157–63. 10.1007/s12028-021-01276-3 34268643

[7] Fisher RS van Emde Boas W Blume W Elger C Genton P Lee P Epileptic seizures and epilepsy: Definitions proposed by the international league against epilepsy (ILAE) and the international bureau for epilepsy (IBE). *Epilepsia.* (2005) 46:470–2. 10.1111/j.0013-9580.2005.66104.x 15816939

[8] Leitinger M Trinka E Gardella E Rohracher A Kalss G Qerama E Diagnostic accuracy of the Salzburg EEG criteria for non-convulsive status epilepticus: a retrospective study. *Lancet Neurol.* (2016) 15:1054–62. 10.1016/S1474-4422(16)30137-5 27571157

[9] Koren J Herta J Draschtak S Pötzl G Pirker S Fürbass F Prediction of rhythmic and periodic EEG patterns and seizures on continuous EEG with early epileptiform discharges. *Epilepsy Behav.* (2015) 49:286–9. 10.1016/j.yebeh.2015.04.044 25982266

[10] Trinka E Cock H Hesdorffer D Rossetti AO Scheffer IE Shinnar S A definition and classification of status epilepticus–report of the ILAE task force on classification of status epilepticus. *Epilepsia.* (2015) 56:1515–23. 10.1111/epi.13121 26336950

[11] Sutter R Dittrich T Semmlack S Rüegg S Marsch S Kaplan PW. Acute systemic complications of convulsive status epilepticus-A systematic review. *Crit Care Med.* (2018) 46:138–45. 10.1097/CCM.0000000000002843 29099419

[12] Jin C Londono I Mallard C Lodygensky GA. New means to assess neonatal inflammatory brain injury. *J Neuroinflammation.* (2015) 12:180. 10.1186/s12974-015-0397-2 26407958 PMC4583178

[13] Payne ET Zhao XY Frndova H McBain K Sharma R Hutchison JS Seizure burden is independently associated with short term outcome in critically ill children. *Brain.* (2014) 137:1429–38. 10.1093/brain/awu042 24595203 PMC3999716

[14] Nasr DM Rabinstein AA. Neurologic complications of extracorporeal membrane oxygenation. *J Clin Neurol.* (2015) 11:383–9. 10.3988/jcn.2015.11.4.383 26320848 PMC4596114

[15] Stockard J Calanchini P Bickford R Billinger T. Electroencephalographic seizures during cardiopulmonary bypass. *J Neurol Neurosurg Psychiatry.* (1974) 37:181–90. 10.1136/jnnp.37.2.181 4819907 PMC494604

[16] Manji RA Grocott HP Manji JS Menkis AH Jacobsohn E. Recurrent seizures following cardiac surgery: Risk factors and outcomes in a historical cohort study. *J Cardiothorac Vasc Anesth.* (2015) 29:1206–11. 10.1053/j.jvca.2015.03.020 26119411

[17] Sharma V Katznelson R Jerath A Garrido-Olivares L Carroll J Rao V The association between tranexamic acid and convulsive seizures after cardiac surgery: a multivariate analysis in 11 529 patients. *Anaesthesia.* (2014) 69:124–30. 10.1111/anae.12516 24588023

[18] Bauer Huang SL Said AS Smyser CD Lin JC Guilliams KP Guerriero RM. Seizures are associated with brain injury in infants undergoing extracorporeal membrane oxygenation. *J Child Neurol.* (2021) 36:230–6. 10.1177/0883073820966917 33112194 PMC8086759

[19] Swartz MF Seltzer LE Cholette JM Yoshitake S Darrow N Algahim MF Intraoperative cortical asynchrony predicts abnormal postoperative electroencephalogram. *Ann Thorac Surg.* (2021) 111:645–54. 10.1016/j.athoracsur.2020.04.090 32511999

[20] Li MY Lou XB Cui YQ Lin RY Ning SY Li LJ Assessment of postoperative risk factors for EEG abnormalities in routine clinical management after paediatric cardiopulmonary bypass. *Interact Cardiovasc Thorac Surg.* (2021) 33:301–8. 10.1093/icvts/ivab081 33822951 PMC8691544

[21] Latal B Wohlrab G Brotschi B Beck I Knirsch W Bernet V. Postoperative amplitude-integrated electroencephalography predicts four-year neurodevelopmental outcome in children with complex congenital heart disease. *J Pediatr.* (2016) 178:55–60.e1. 10.1016/j.jpeds.2016.06.050 27453368

[22] Gaynor JW Jarvik GP Gerdes M Kim DS Rajagopalan R Bernbaum J Postoperative electroencephalographic seizures are associated with deficits in executive function and social behaviors at 4 years of age following cardiac surgery in infancy. *J Thorac Cardiovasc Surg.* (2013) 146:132–7. 10.1016/j.jtcvs.2013.04.002 23768805 PMC4617776

[23] Tschernatsch M Juenemann M Alhaidar F El Shazly J Butz M Meyer M Epileptic seizure discharges in patients after open chamber cardiac surgery—a prospective prevalence pilot study using continuous electroencephalography. *Intensive Care Med.* (2020) 46:1418–24. 10.1007/s00134-020-06073-8 32405742 PMC7334279

[24] Koster A Börgermann J Zittermann A Lueth JU Gillis-Januszewski T Schirmer U. Moderate dosage of tranexamic acid during cardiac surgery with cardiopulmonary bypass and convulsive seizures: incidence and clinical outcome. *Br J Anaesth.* (2013) 110:34–40. 10.1093/bja/aes310 22986419

[25] Goldstone AB Bronster DJ Anyanwu AC Goldstein MA Filsoufi F Adams DH Predictors and outcomes of seizures after cardiac surgery: a multivariable analysis of 2,578 patients. *Ann Thorac Surg.* (2011) 91:514–8. 10.1016/j.athoracsur.2010.10.090 21256303

[26] Seltzer LE Swartz M Kwon JM Burchfiel J Alfieris GM Guillet R. Intraoperative electroencephalography predicts postoperative seizures in infants with congenital heart disease. *Pediatr Neurol.* (2014) 50:313–7. 10.1016/j.pediatrneurol.2013.12.017 24507699 PMC4203305

[27] Raja R Johnston JK Fitts JA Bailey LL Chinnock RE Ashwal S. Post-transplant seizures in infants with hypoplastic left heart syndrome. *Pediatr Neurol.* (2003) 28:370–8. 10.1016/S0887-8994(03)00018-3 12878299

[28] Beyea MM Tillmann BW Iansavichene AE Randhawa VK Van Aarsen K Nagpal AD. Neurologic outcomes after extracorporeal membrane oxygenation assisted CPR for resuscitation of out-of-hospital cardiac arrest patients: A systematic review. *Resuscitation.* (2018) 130:146–58. 10.1016/j.resuscitation.2018.07.012 30017957

[29] Ong CS Etchill E Dong J Shou BL Shelley L Giuliano K Neuromonitoring detects brain injury in patients receiving extracorporeal membrane oxygenation support. *J Thorac Cardiovasc Surg.* (2021):S0022-5223(21)01508-7. 10.1016/j.jtcvs.2021.09.063 34865837

[30] Cho SM Choi CW Whitman G Suarez JI Martinez NC Geocadin RG Neurophysiological findings and brain injury pattern in patients on ECMO. *Clin EEG Neurosci.* (2021) 52:462–9. 10.1177/1550059419892757 31823652

[31] Cvetkovic M Chiarini G Belliato M Delnoij T Zanatta P Taccone FS International survey of neuromonitoring and neurodevelopmental outcome in children and adults supported on extracorporeal membrane oxygenation in Europe. *Perfusion.* (2021) :2676591211042563. 10.1177/02676591211042563 34550013

[32] Bembea MM Felling R Anton B Salorio CF Johnston MV. Neuromonitoring during extracorporeal membrane oxygenation: A systematic review of the literature. *Pediatr Crit Care Med.* (2015) 16:558–64. 10.1097/PCC.0000000000000415 25828783

[33] Cook RJ Rau SM Lester-Pelham SG Vesper T Peterson Y Adamowski T Electrographic seizures and brain injury in children requiring extracorporeal membrane oxygenation. *Pediatr Neurol.* (2020) 108:77–85. 10.1016/j.pediatrneurol.2020.03.001 32299743

[34] Cho SM Ziai W Mayasi Y Gusdon AM Creed J Sharrock M Noninvasive neurological monitoring in extracorporeal membrane oxygenation. *ASAIO J.* (2020) 66:388–93. 10.1097/MAT.0000000000001013 31045914

[35] Said AS Guilliams KP Bembea MM. Neurological monitoring and complications of pediatric extracorporeal membrane oxygenation support. *Pediatr Neurol.* (2020) 108:31–9. 10.1016/j.pediatrneurol.2020.03.014 32299748 PMC7698354

[36] Fuster RG Argudo JA Albarova OG Sos FH López SC Codoñer MB Prognostic value of chronic obstructive pulmonary disease in coronary artery bypass grafting. *Eur J Cardiothorac Surg.* (2006) 29:202–9. 10.1016/j.ejcts.2005.11.015 16376093

[37] De Reuck J Proot P Van Maele G. Chronic obstructive pulmonary disease as a risk factor for stroke-related seizures. *Eur J Neurol.* (2007) 14:989–92. 10.1111/j.1468-1331.2007.01829.x 17718690

[38] Helmers SL Wypij D Constantinou JE Newburger JW Hickey PR Carrazana EJ Perioperative electroencephalographic seizures in infants undergoing repair of complex congenital cardiac defects. *Electroencephalogr Clin Neurophysiol.* (1997) 102:27–36. 10.1016/S0013-4694(96)95079-8 9060852

[39] Kochanek M Kochanek J Böll B Eichenauer DA Beutel G Bracht H Veno-venous extracorporeal membrane oxygenation (vv-ECMO) for severe respiratory failure in adult cancer patients: a retrospective multicenter analysis. *Intensive Care Med.* (2022) 48:332–42. 10.1007/s00134-022-06635-y 35146534 PMC8866383

[40] Manickavel S. Pathophysiology of respiratory failure and physiology of gas exchange during ECMO. *Indian J Thorac Cardiovasc Surg.* (2021) 37:203–9. 10.1007/s12055-020-01042-8 33967443 PMC8062645

[41] Lin JC. Extracorporeal membrane oxygenation for severe pediatric respiratory failure. *Respir Care.* (2017) 62:732–50. 10.4187/respcare.05338 28546375

[42] Kozinn J Wrisinger WC. ECMO for adults with severe respiratory failure. *Mo Med.* (2019) 116:58–62. 30862988 PMC6390783

[43] Feldhaus D Brodie D Lemaitre P Sonett J Agerstrand C. The evolution of the use of extracorporeal membrane oxygenation in respiratory failure. *Membranes.* (2021) 11:491. 10.3390/membranes11070491 34208906 PMC8305045

[44] Grant C Jr. Richards JB Frakes M Cohen J Wilcox SR. ECMO and right ventricular failure: Review of the literature. *J Intensive Care Med.* (2021) 36:352–60. 10.1177/0885066619900503 31964208

[45] Wild KT Rintoul N Kattan J Gray B. Extracorporeal life support organization (ELSO): Guidelines for neonatal respiratory failure. *ASAIO J.* (2020) 66:463–70. 10.1097/MAT.0000000000001153 32282347

[46] Peluso L Rechichi S Franchi F Pozzebon S Scolletta S Brasseur A Electroencephalographic features in patients undergoing extracorporeal membrane oxygenation. *Crit Care.* (2020) 24:629. 10.1186/s13054-020-03353-z 33126887 PMC7598240

[47] Shellhaas RA. Seizure classification, etiology, and management. *Handb Clin Neurol.* (2019) 162:347–61. 10.1016/B978-0-444-64029-1.00017-5 31324320

[48] Lou X Liu Y Cui Y Li J Li L Ma L Contemporary trends and risk factors of hemodynamic and myocardial mechanics derived by the pressure recording analytical method after pediatric cardiopulmonary bypass. *Front Cardiovasc Med.* (2021) 8:687150. 10.3389/fcvm.2021.687150 34355027 PMC8330813

[49] Sakamoto T. Current status of brain protection during surgery for congenital cardiac defect. *Gen Thorac Cardiovasc Surg.* (2016) 64:72–81. 10.1007/s11748-015-0606-z 26620539

[50] Kiziltug H Falter F. Circulatory support during lung transplantation. *Curr Opin Anaesthesiol.* (2020) 33:37–42. 10.1097/ACO.0000000000000806 31714270

[51] Pataraia E Jung R Aull-Watschinger S Skhirtladze-Dworschak K Dworschak M. Seizures after adult cardiac surgery and interventional cardiac procedures. *J Cardiothorac Vasc Anesth.* (2018) 32:2323–9. 10.1053/j.jvca.2017.12.036 29398383

[52] Hanif S Sinha S Siddiqui KA. Electroencephalography findings in patients with acute post coronary artery bypass graft encephalopathy. *Neurosciences.* (2014) 19:331–3. 25274597 PMC4727676

