# Supplementary material for: Incidence, risk factors, and outcomes in electroencephalographic seizures after mechanical circulatory support: A systematic review and meta-analysis
Source: Front Cardiovasc Med. 2022 Aug 3;9:872005. doi: 10.3389/fcvm.2022.872005 (PMC9381842; doi:10.3389/fcvm.2022.872005)
Supplement: Supplementary File 1 — Search strategy. [file Data_Sheet_1.PDF]

Pubmed: Final result #8

#8 ••• >

Search: **((((((Electroencephalography[MeSH Terms]) OR (Electroencephalography[MeSH Major Topic])) OR (Electroencephalography)) OR (EEG)) OR (Electroencephalograms)) AND ((((Seizure[MeSH Terms]) OR (Seizure[MeSH Major Topic])) OR (Seizure*)) OR (epileps*))) AND ((((((((((((Cardiopulmonary Bypass[MeSH Terms]) OR (Cardiopulmonary Bypass[MeSH Major Topic]))) OR (Heart-Lung Bypass)) OR (Bypass, Heart-Lung)) OR (Bypasses, Heart-Lung)) OR (Heart Lung Bypass)) OR (Heart-Lung Bypasses)) OR (Bypass, Cardiopulmonary)) OR (Bypasses, Cardiopulmonary)) OR (Cardiopulmonary Bypasses)) OR ((((((((((((((((((((((Extracorporeal Membrane Oxygenation[MeSH Terms]) OR (Extracorporeal Membrane Oxygenation[MeSH Major Topic])) OR (Extracorporeal Membrane Oxygenations)) OR (Membrane Oxygenation, Extracorporeal)) OR (Oxygenation, Extracorporeal Membrane)) OR (ECMO Treatment)) OR (ECMO Treatments)) OR (Treatment, ECMO)) OR (ECLS Treatment)) OR (ECLS Treatments)) OR (Treatment, ECLS)) OR (ECMO Extracorporeal Membrane Oxygenation)) OR (Extracorporeal Life Support)) OR (Extracorporeal Life Supports)) OR (Life Support, Extracorporeal)) OR (Venoarterial ECMO)) OR (ECMO, Venoarterial)) OR (Venoarterial ECMOs)) OR (Venoarterial Extracorporeal Membrane Oxygenation)) OR (Venovenous ECMO)) OR (ECMO, Venovenous)) OR (Venovenous ECMOs)) OR (Venovenous Extracorporeal Membrane Oxygenation)))**

Embase: Final result #15

History | Save | Delete | Print view | Export | Email | Combine > | using ● And ○ Or

#15	#13 AND #14
#14	#3 AND #6
#13	#9 OR #12
#12	#10 OR #11
#11	**'extracorporeal membrane oxygenation*'** OR **'ecmo treatment*'** OR **'ecls treatment*'** OR **'extracorporeal life support*'** OR **'venoarterial ecmo*'** OR **'venoarterial extracorporeal membrane oxygenation'** OR **'venovenous ecmo*'** OR **'venovenous extracorporeal membrane oxygenation'**
#10	**'extracorporeal oxygenation'**/exp
#9	#7 OR #8
#8	**'cardiopulmonary bypass*'** OR **'heart lung bypass*'**
#7	**'cardiopulmonary bypass'**/exp
#6	#4 OR #5
#5	**'electroencephalogram*'** OR **'eeg'**
#4	**'electroencephalography'**/exp
#3	#1 OR #2
#2	**seizure*** OR **epileps***
#1	**'seizure'**/exp

CENTRAL :final results #33

#21	(Seizure* OR epileps*)<br>(Word variations have been searched)	S	Limits
#22	#20 OR #21		Limits
#23	MeSH descriptor: [Electroencephalography] explode all trees	MeSH	
#24	(Electroencephalogram* OR EEG)<br>(Word variations have been searched)	S	Limits
#25	#23 OR #24		Limits
#26	MeSH descriptor: [Cardiopulmonary Bypass] explode all trees	MeSH	
#27	(cardiopulmanary bypass* OR heart-lung bypass*)<br>(Word variations have been searched)	S	Limits
#28	#26 OR #27		Limits
#29	MeSH descriptor: [Extracorporeal Membrane Oxygenation] explode all trees	MeSH	
#30	('Extracorporeal Membrane Oxygenation*' OR 'ECMO Treatment*' OR 'ECLS Treatment' OR 'Extracorporeal Life Support*' OR 'Venoarterial ECMO*' OR 'Venoarterial Extracorporeal Membrane Oxygenation' OR 'Venovenous ECMO*' OR 'Venovenous Extracorporeal Membrane Oxygenation')<br>(Word variations have been searched)	S	Limits
#31	#29 OR #30		Limits
#32	#28 OR #31		Limits
#33	#22 AND #25 AND #32		Limits
