# Supplementary material for: Incidence, risk factors, and outcomes in electroencephalographic seizures after mechanical circulatory support: A systematic review and meta-analysis
Source: Front Cardiovasc Med. 2022 Aug 3;9:872005. doi: 10.3389/fcvm.2022.872005 (PMC9381842; doi:10.3389/fcvm.2022.872005)
Supplement: Supplementary File 2 — Risk of bias in enrolled studies. [file Data_Sheet_2.pdf]

All studies included are cohort studies, we thus referred to NOS scale for quality assessment. While the NOS scale is used for studies with a non-exposed cohort, which was not the case for our study aiming to summarize Electroencephalography(EEG) seizures incidence, we made several modifications to the scale. The modified NOS scale is as follow.

Selection（3 stars maximum）

1） Representativeness of the exposed cohort
2） Ascertainment of exposure
3） Demonstration that outcome of interest was not present at start of the study

Outcome（3 stars maximum）

1） Assessment of outcome
2） Was follow-up long enough for outcomes to occur
3） Adequacy of follow up of cohorts

Selection

1) Representativeness of the exposed cohort

a) truly representative of the average _______________ (describe) in the community ※

b) somewhat representative of the average ______________ in the community ※

c) selected group of users eg nurses, volunteers

d) no description of the derivation of the cohort

2) Ascertainment of exposure

a) secure record (eg mechanical support records) ※

b) structured interview ※

c) written self-report

d) no description

3) Demonstration that outcome of interest was not present at start of study

a) yes ※

b) no

Outcome

1) Assessment of outcome

a) independent assessesd by electroencephalography ※

b) record linkage ※

c) self -report

d) no description

2) Was follow-up long enough for outcomes to occur

a) yes (select an adequate follow up period for outcome of interest) ※

b) no

3) Adequacy of follow up of cohorts

a) complete follow up - all subjects accounted for ※

b) subjects lost to follow up unlikely to introduce bias - small number lost - > ____ % (select an adequate %) follow up, or description provided of those lost)

c) follow up rate < ____% (select an adequate %) and no description of those lost

d) no statement

Supplement Table1 Risk of bias——Adult

	Selection			Outcome		
	Representativeness	Ascertainment of exposure	Outcome was not present before	Assessment	Follow-up long enough	Adequacy of follow up of cohorts
Gofton, T. E., et al. (2014)	⭐	⭐ (CPB)	⭐	⭐	⭐	0
Goldstone, A. B., et al. (2011)	⭐	⭐ (CPB)	⭐	0(clinically or EEG)	⭐	0
Hanif, S., et al. (2014).	⭐	⭐	⭐	⭐	0	0
Koster, A., et al. (2013)	⭐	⭐ (CPB)	⭐	0	0	0
Mani, R., et al. (2012)	⭐	⭐	⭐	⭐	0	0
Manji, R. A., et al. (2015)	⭐	⭐ (CPB)	⭐	⭐	⭐	0
Marcuse, L. V., et al. (2014)	⭐	⭐ (CPB)	⭐	⭐	⭐	0(no statement)
Sharma, V., et al. (2014)	⭐	⭐ (CPB)	⭐	0	0	0
Stockard, J., et al. (1974)	0	⭐ (CPB)	⭐	⭐	0	0
Tschernatsch, M., et al. (2020)	⭐	⭐ (CPB)	⭐	⭐	⭐	0
Cabral, P., et al. (2020)	⭐	⭐ (ECMO)	⭐	⭐	⭐	0
Nasr, D. M. and A. A. Rabinstein (2015)	⭐	⭐ (ECMO)	⭐	0	0	0
Peluso, L. (2020)	⭐	⭐ (ECMO)	⭐	⭐	⭐	⭐

Supplement Table2 Risk of bias——Pediatric

	Selection			Outcome		
	Representativeness	Ascertainment of exposure	Outcome was not present before	Assessment	Follow-up long enough	Adequacy of follow up of cohorts
Agha-2021	⭐（all children and infants）	0(not all use CPB)	⭐	0(24/38 with seizures use EEG)	⭐ (3 months)	0(not all people are followed up)
Bellinger-1999	⭐ (all infants)	⭐ (cardiac arrest or CPB)	⭐	0(clinically or EEG)	⭐	0(158/163)
helmers1997	⭐ (infants)	⭐ (cardiac arrest or CPB)	⭐	⭐	0	0
Newburger-1993	⭐	⭐ (cardiac arrest or CPB)	⭐	⭐	0	0
Gui, J., et al. (2020).	⭐ (infants)	⭐ (CPB)	⭐	⭐	⭐	⭐
Gunn, J. K., et al. (2012).	⭐ (neonates)	⭐ (CPB)	⭐	⭐	⭐	0(2 had been lost to follow-up)
Latal, B., et al. (2016).	⭐ (infants)	⭐ (CPB)	⭐	⭐	⭐	0
Levy, R. J., et al. (2021).	⭐ (infants)	⭐	⭐	⭐	0	0
Li, M. Y., et al. (2021).	⭐	⭐ (CPB)	⭐	⭐	0	0
Andropoulos, D. B., et al. (2010).	⭐	⭐ (CPB)	⭐	⭐	0	0

Claessens, N. H. P., et al. (2018).	⭐	⭐ (CPB)	⭐	0 (they used 2-channel aEEG not the standard one)	0	0
Clancy, R. R., et al. (2005).	0(English spoken only)	⭐ (CPB)	⭐	⭐	0	0
Du Plessis, A. J., et al. (1997).	⭐ (infants)	⭐ (CPB)	⭐	0(clinically or EEG)	0	0
Gunn, J. K., et al. (2012).	⭐	0(CPB used in 83%)	⭐	⭐ (they use two-channel aEEG instead of conventional multichannel EEG)	⭐	0
Gaynor, J. W., et al. (2006).	0(all children but a language other than English spoken in the home were excluded)	⭐ (all use CPB and some use DHCA)	⭐	⭐	⭐	0
Gaynor, J. W., et al. (2013).	0(all children but a language other than English spoken in the home were excluded)	⭐ (all use CPB and some use DHCA)	⭐	⭐	⭐	0
Limperopoulos, C., et al. (2001).	⭐	⭐ (CPB)	⭐	⭐	⭐	0
Prajongkit, T., et al. (2019).	⭐	0(therapeutical hypothermia)	⭐	⭐	⭐	⭐

Naim, M. Y., et al. (2015).	★	★ (CPB)	★	★	0	0
Raja, R., et al. (2003)	★	★ (CPB)	★	0(The diagnosis of seizures was made clinically and based on the observations of the nursing or medical staff witnessing a seizure.)	★	0
Rappaport, L. A., et al. (1998).	★	★ (CPB)	★	0(136/171 were monitored on EEG)	★	0
Seltzer, L. E., et al. (2014).	★	★ (CPB,17/32 DHCA)	★	★	0	0
Stockard, J., et al. (1974).	0	★ (CPB)	★	★	0	0
Swartz, M. F., et al. (2021).	★	★ (CPB)	★	★	0	0
Turner, II, et al. (2021)	★	★ (cardiac arrest or CPB)	★	★	0	0
Bauer Huang, S. L., et al. (2021)	★ (all infants)	★ (ECMO)	★	0(Total:104; Continuous EEG:45; Without EEG:59; Seizure:18/45)	0	0
Chahine, A., et al. (2021)	★ (neonates and	★ (ECMO)	★	★	0	0

	Children)					
Hassumani, D. O., et al. (2021).	⭐ (children)	⭐ (ECMO)	⭐	⭐ (93% received EEG)	0	0
Hervey-Jumper, S. L., et al. (2011)	⭐ (26529children and 1170 adults)	⭐ (ECMO)	⭐	0(clinical seizure:8.4%; EEG confirmed seizure:2.1%)	⭐	0
Horan, M., et al. (2007)	⭐ (neonates)	⭐	⭐	⭐	0	0
LaRovere, K. L., et al. (2017)	⭐ (children)	⭐ (ECMO)	⭐	0(36% underwent EEG monitoring)	0	0
Nasr, D. M. and A. A.	0	⭐ (ECMO)	⭐	0(no statement)	0	0
Ostendorf, A. P., et al. (2016).	⭐ (children)	0(not all use ECMO)	⭐	⭐	0	0
Polito, A., et al. (2013).	⭐ (neonates)	⭐ (ECMO)	⭐	0(clinically or EEG)	0	0
Cammock, C., et al. (2018).	⭐	⭐ (ECMO)	⭐	0((including witnessed clinical seizure or subclinical noted on EEG)	⭐	⭐
Catherine M. Gannon, M., et al. (2001)	⭐	⭐ (ECMO)	⭐	⭐	0	0
Cook, R. J., et al. (2020).	0(neonates to age 21 years)	⭐ (ECMO)	⭐	⭐	0	0
Dimmitt, R. A., et al. (2001).	⭐	⭐ (ECMO)	⭐	0(no statement of EEG seizure)	0	0
Fox, J., et al. (2020).	⭐	⭐ (ECMO)	⭐	⭐	0	0
Graziani, L. J., et al. (1994).	⭐	⭐	⭐	⭐	⭐	⭐

		(ECMO)				
Hahn, J. S., et al. (1993).	⭐	⭐ (ECMO)	⭐	⭐	⭐	0
Lin, J. J., et al. (2017).	⭐	⭐ (ECMO)	⭐	0(99/112 used cEEG)	0	0
Okochi, S., et al. (2018).	⭐	⭐ (ECMO)	⭐	⭐	0	0
Piantino, J. A., et al. (2013).	⭐ (children)	⭐ (Extracorporeal Cardiac Life Support)	⭐	⭐ (19/49 used EEG monitoring)	0	0
Sansevere, A. J., et al. (2020).	⭐	⭐ (ECMO)	⭐	⭐	0	0
Streletz, L. J., et al. (1992).	⭐	⭐ (ECMO)	0(67/145 used preECMO EEG)	0(EEG and/or clinical)	⭐	0
Yuliati, A., et al. (2020).	⭐	⭐ (ECMO)	⭐	⭐	⭐	0(7/29 lost follow-up)
